# Skeletal Fluorosis: An Unusual Manifestation of Computer Cleaner Inhalant Abuse

**DOI:** 10.7759/cureus.8461

**Published:** 2020-06-05

**Authors:** Billy-Joe Liane, Annie Chow, David Kline

**Affiliations:** 1 Internal Medicine, Brooke Army Medical Center, San Antonio, USA; 2 Anesthesiology, Brooke Army Medical Center, San Antonio, USA

**Keywords:** skeletal fluorosis, inhalant abuse, osteosclerosis

## Abstract

Skeletal fluorosis is a metabolic bone disease caused by accumulation of fluoride and is generally associated with chronic exposure to fluoride-contaminated groundwater, a phenomenon endemic to developing countries. Whereas elevated water fluoride concentrations do not constitute a public health issue in the United States, emergence of skeletal fluorosis as a sequela of chronic recreational exposures has been described. In this case report, our 33-year-old male patient with a history of major depressive disorder and substance abuse was hospitalized for hyperkalemia and acute kidney injury discovered on routine bloodwork due to concomitant nonsteroidal anti-inflammatory drugs (NSAID) and antihypertensive use. Upon hospital admission, he was found to be anemic with a significantly elevated alkaline phosphatase. Given a history of low back pain in the setting of these laboratory abnormalities, lower spine and pelvic imaging revealed diffusely increased bone density and sclerosis. Hematologic evaluation ensued to include a peripheral smear and bone marrow biopsy. Given the patient’s history of computer cleaner inhalant abuse, serum and urinary fluoride levels were obtained. Serum fluoride returned within normal limits though urinary fluoride was increased. Bone marrow histopathology revealed prominent diffuse sclerosis which in conjunction with urinary fluoride levels and computer cleaner inhalant abuse history supported the diagnosis of skeletal fluorosis. Skeletal fluorosis in the United States is rare and presents with non-specific findings requiring a high index of suspicion based on a detailed patient history for expedient diagnosis.

## Introduction

Skeletal fluorosis, a rare clinical entity in developed nations, is endemic to many developing countries around the world, most notably in China and India, where it is caused by chronic ingestion of fluoride-concentrated groundwater [1]. However, in recent years in the United States skeletal fluorosis has been described as a result of chronic recreational substance abuse with volatile anesthetics, fluoride-containing aerosolized computer cleaner, and surreptitious toothpaste ingestion [2].

## Case presentation

We present a case of a 33-year-old male with significant psychiatric history including major depressive disorder and computer cleaner inhalant abuse who was admitted from an inpatient psychiatric facility for hyperkalemia and acute kidney injury. The morning of his hospital admission, he was evaluated by an endocrinologist for suspected metabolic bone disease in the setting of chronic low back pain and approximately three years of uptrending alkaline phosphatase (ALP) up to 780 U/L. Roentgenography of the lower spine and pelvis obtained approximately two months prior to presentation revealed osteoblastic lesions concerning for Paget’s disease. Routine metabolic evaluation revealed hypovitaminosis D (12 ng/mL) and elevated parathyroid hormone (101 pg/mL; ref. range: 10-65 pg/mL) with suspicion for osteomalacia as the etiology of his elevated ALP. Metabolic bone disease evaluation otherwise was unremarkable.

However, routine laboratory evaluation obtained following his endocrinology appointment revealed a serum potassium of 6.6 mEq/L and creatinine of 2.2 mg/dL due to concomitant use of prescribed lisinopril, spironolactone, ibuprofen and meloxicam. He was admitted for further management. Upon admission, the patient endorsed no acute symptoms, but he did endorse chronic musculoskeletal pain, particularly of the lower back. Other past medical history included hypertension, dyslipidemia, chronic low back pain and constipation.

**Figure 1:**
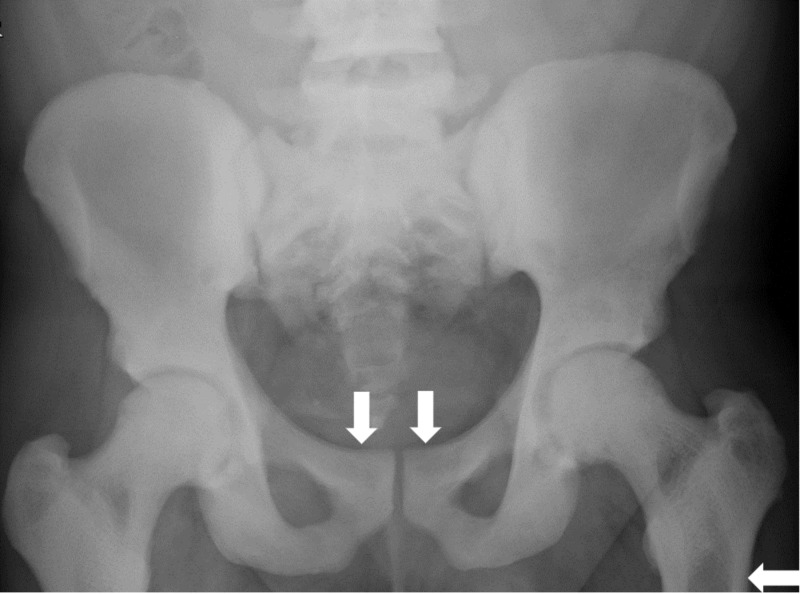
Pelvic radiograph Diffuse increased density of visible bones with smooth, symmetric periosteal reaction along the superior pubic rami and subtrochanteric femurs.

**Figure 2:**
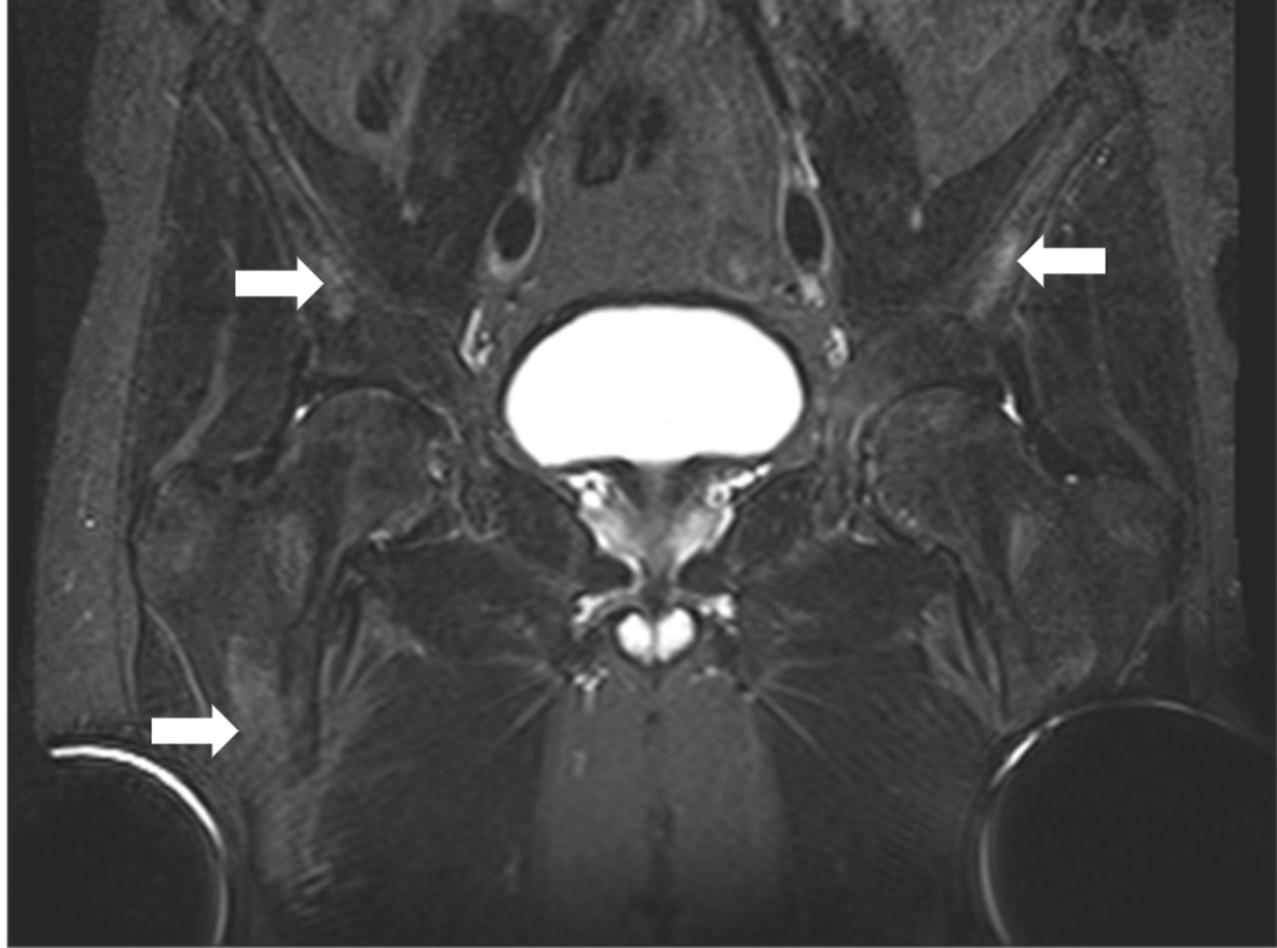
Pelvic magnetic resonance imaging Diffuse short tau inversion recovery (STIR) hyperintensities throughout the osseous pelvis concerning for metabolic bone disease and possible osteonecrosis.

His hyperkalemia was treated upon admission and the patient’s home anti-hypertensives and nonsteroidal anti-inflammatory drugs (NSAIDs) were held in the setting of acute kidney injury. He was hydrated and renal function and electrolytes normalized. He was also found to be newly anemic with a hemoglobin of 9 g/dL. After development of new onset hip pain during hospitalization, X-rays were obtained with subsequent magnetic resonance imaging (MRI) revealing diffusely increased density of osseous structures (Figures 1, 2).

Given anemia, elevated ALP and increased bone density on imaging, an inpatient hematology/oncology consultation was obtained. Peripheral smear and his reticulocyte index were consistent with hypoproliferation, and bone marrow biopsy was subsequently performed. Urine and serum protein electrophoresis and urine light chains were not consistent with multiple myeloma.

**Figure 3:**
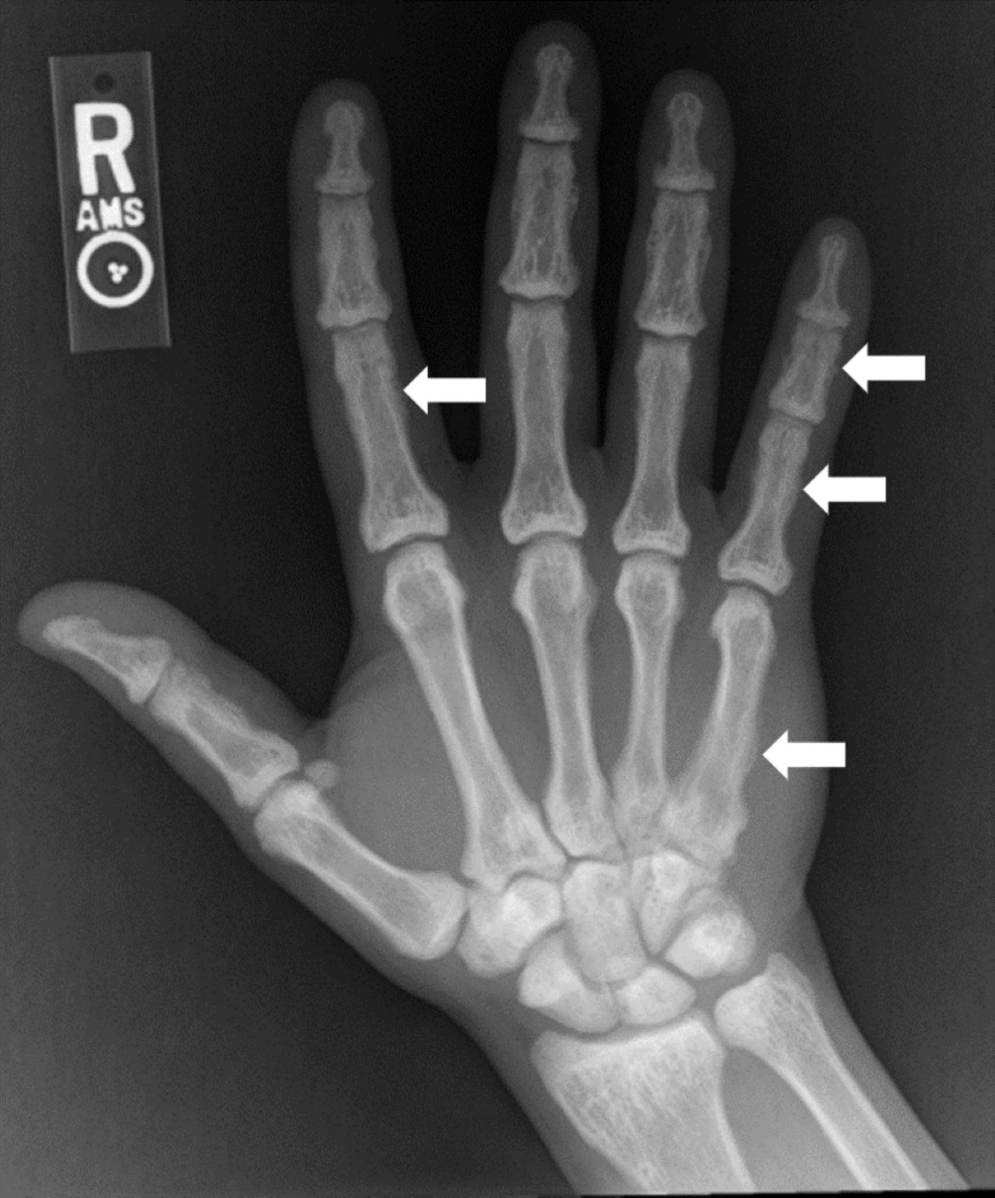
Right hand/wrist radiograph Diffuse sclerosis with multiple areas of indistinct cortex/periosteal reaction, most prominently involving the 2nd and 5th digits and 5th metacarpal.

With the patient’s history of chronic computer cleaner inhalant abuse and elevated 1-difluoroethane levels (20 mcg/mL) obtained several months prior to presentation (obtained to monitor for inhalant abuse), we performed a literature review to determine if possible relationships between skeletal abnormalities and computer cleaner inhalant abuse have been noted. After reviewing rare case reports associating inhaled fluorine-containing compounds and skeletal fluorosis, serum and urinary fluoride levels were obtained as well as extremity X-rays to look for further evidence of possible skeletal fluorosis (Figure 3). Of note, hand X-rays from 2016 revealed no sclerosis.

**Figure 4:**
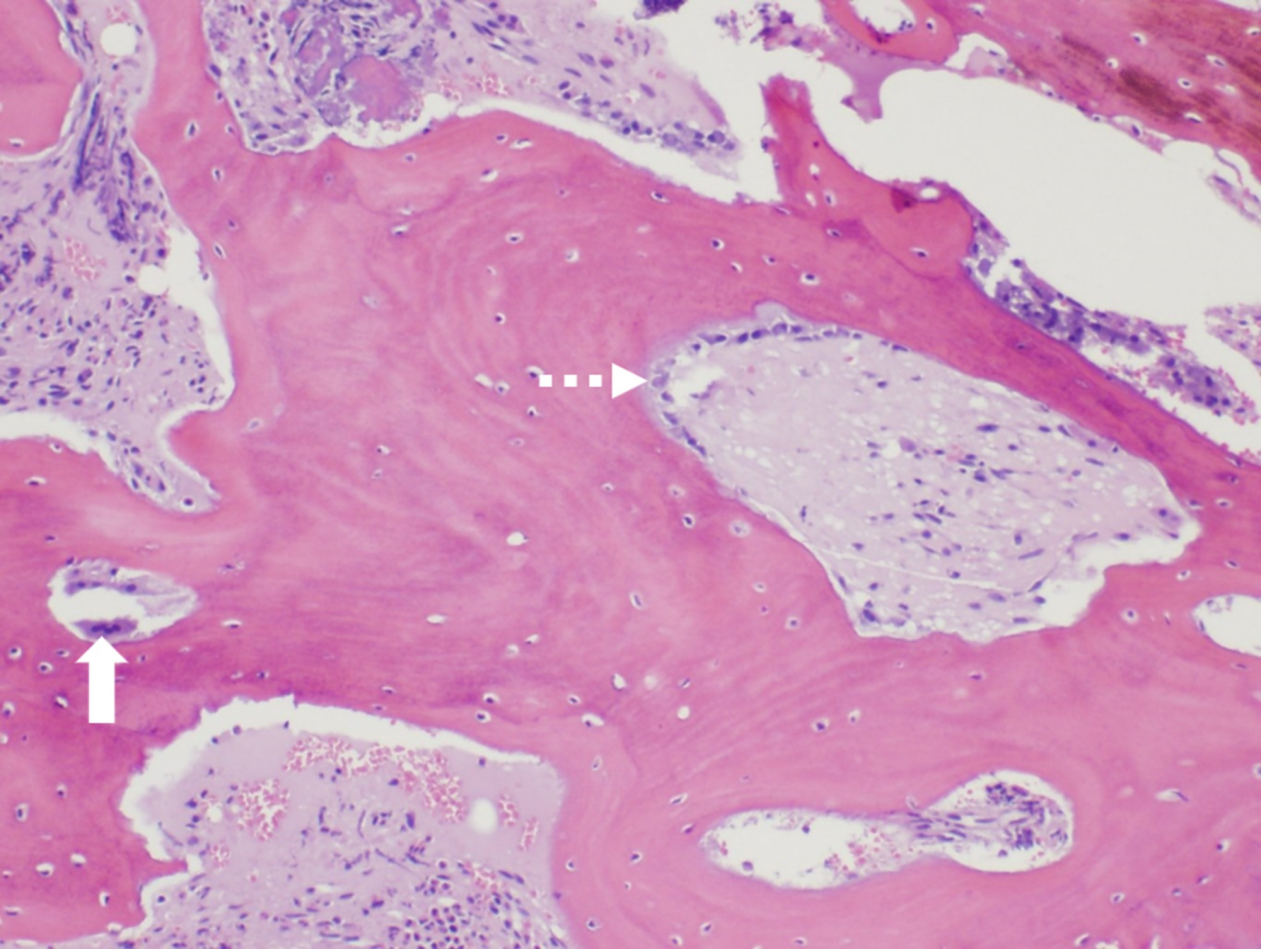
Bone marrow biopsy 100x hematoxylin and eosin stained robust and thickened bone demonstrating osteoclast activity (solid arrow) and osteoblast activity (dashed arrow) consistent with osteosclerosis.

Bone marrow biopsy revealed prominent cortical bone initially concerning for tangential biopsy and inadequate sampling. After discussing with the pathologist the patient’s history and verifying adequate sampling, it was felt the biopsy was consistent with osteosclerosis due to skeletal fluorosis with possible encroachment of bone to his bone marrow likely leading to his hypoproliferative anemia (Figure 4).

The serum fluoride level was within normal limits. Interestingly, urine fluoride was significantly elevated at 19 mg/L (ref. range: 0.2-3.2 mg/L) and the fluoride to creatinine ratio was significantly elevated (27.5 mg/g, upper limit of normal 3.0), rendering skeletal fluorosis the most likely diagnosis based on the patient’s history, presentation, imaging and laboratory findings. No other etiology for his skeletal abnormalities and elevated ALP was found.

The patient was transferred back to the inpatient psychiatric facility after recovering from the acute kidney injury and the hyperkalemia. Approximately six months after this hospitalization, his ALP had decreased to 372 U/L with continued inhalant sobriety. However, he had not yet followed up with an endocrinologist for continued outpatient care.

## Discussion

Skeletal fluorosis in the United States is rare and presents with non-specific findings such as back pain and elevated ALP, requiring a high index of suspicion based on detailed patient history for expedient diagnosis and minimization of unnecessary testing. Its inclusion in the differential for skeletal pain and diffuse osteosclerosis would help clue clinicians to further prompt patients about means of exposure to fluoride and obtain confirmatory studies.

Osteosclerosis on imaging and bone biopsy are consistent with the disease. Secondary hyperparathyroidism is also frequently noted [3,4]. While elevated serum, urine, and bone fluoride levels are usually seen, urinary fluoride levels can remain elevated despite normal serum levels following cessation of inhalant abuse, as occurred in our case (the patient had refrained for two months prior to presentation due to psychiatric hospitalization) [5-7].

Quantitative bone ash fluoride analysis from a bone biopsy is considered the gold standard for definitive diagnosis [7]. Additional diagnostic approaches include dual-energy X-ray absorptiometry (DXA) which can show elevated Z-scores as noted in previous publications [7]. The increase in bone mineral density is thought to be related to fluoride’s enhanced osteoblastic activity. Careful interpretation of DXA scans is warranted in skeletal fluorosis patients since the disorganized osseous remodeling process in skeletal fluorosis may place them at an increased fracture risk [7].

Elimination of excessive ingestion is the initial and most important intervention in the management of skeletal fluorosis followed by restoration of vitamin D levels. Maintaining proper hydration is also recommended since nephrolithiasis and hypercalciuria have been described during skeletal unloading of fluoride [7].

## Conclusions

Limited data is available regarding management and progression of skeletal fluorosis secondary to the inhalation of computer cleaners, given the paucity of these particular cases. As awareness of skeletal fluorosis increases, both time to diagnosis and prevalence will likely decrease with the help of reliable diagnostic tools and better characterization of the association of skeletal findings with fluorosis.

## References

[1] Krishnamachari KA (1986). Skeletal fluorosis in humans: a review of recent progress in the understanding of the disease. Prog Food Nutr Sci.

[2] Kurland ES, Schulman RC, Zerwekh JE, Reinus WR, Dempster DW, Whyte MP (2007). Recovery from skeletal fluorosis (an enigmatic, American case). J Bone Miner Res.

[3] Peicher K, Maalouf NM (2017). Skeletal fluorosis due to fluorocarbon inhalation from an air dust cleaner. Calcif Tissue Int.

[4] Koroglu BK, Ersoy IH, Koroglu M, Balkarli A, Ersoy S, Varol S, Tamer MN (2011). Serum parathyroid hormone levels in chronic endemic fluorosis. Biol Trace Elem Res.

[5] Tucci JR, Whitford GM, McAlister WH, Novack DV, Mumm S, Keaveny TM, Whyte MP (2017). Skeletal fluorosis due to inhalation abuse of a difluoroethane-containing computer cleaner. J Bone Mine Res.

[6] Ponce A, Oakes JA, Eggleston W (2020). Acute skeletal fluorosis in the setting of 1,1-difluoroethane abuse. Clin Toxicol.

[7] Cohen E, Hsu RY, Evangelista P, Aaron R, Rubin LE (2014). Rapid-onset diffuse skeletal fluorosis from inhalant abuse: a case report. J Bone Joint Surg Case Connect.

